# Moderate Stability among Delay, Probability, and Effort Discounting in Humans

**DOI:** 10.1007/s40732-023-00537-1

**Published:** 2023-02-15

**Authors:** Gisel G. Escobar, Silvia Morales-Chainé, Jeremy M. Haynes, Carlos Santoyo, Suzanne H. Mitchell

**Affiliations:** 1 Faculty of Psychology, National Autonomous University of Mexico, Universidad Avenue 3004, Coyoacán, 04510 Mexico City, Mexico; 2 Faculty of Higher Studies Iztacala, National Autonomous University of Mexico, State of Mexico, Mexico; 3 Utah State University, Logan, UT USA; 4 Oregon Health & Science University, Portland, OR USA

**Keywords:** Delay discounting, Effort discounting, Probability discounting, Stability, Mathematical functions

## Abstract

The stability of delay discounting across time has been well-established. However, limited research has examined the stability of probability discounting, and no studies of the stability of effort discounting are available. The present study assessed the steady-state characteristics of delay, probability, and effort discounting tasks across time with hypothetical rewards in humans, as well as whether response characteristics suggested a common discounting equation. Participants completed delay, probability, and effort discounting tasks on three occasions. We found moderate relative stability of delay and probability tasks, and similar evidence for absolute stability across time for all tasks. The interclass correlations coefficient showed some correspondence across time points and tasks, and higher levels of between subject variability, especially for the effort discounting task, suggesting trait level variables has a stronger influence on performance than state level variables. Performance on the delay and probability tasks were moderately correlated and similar mathematical functions fit choice patterns on both tasks (hyperbolic), suggesting that delay and probability discounting processes shared some common elements. Lower correlations and different function fits suggested that effort discounting involves more unique features.

Delay discounting is the process by which an outcome loses value as the delay to its receipt increases and is widely used to describe intertemporal choices in human and nonhuman animals (Odum, 2011; Rachlin et al., 1991). Measures of delay discounting in humans typically involve assessing preferences between hypothetical outcomes that vary in amount and delay. Steep delay discounting reflects a relative preference for smaller, sooner rewards (i.e., more impatience), and has been called a trans-disease process because of its association with many significant health problems (see Amlung et al., 2017; Bickel et al., 2012).

Delay can be considered a *cost* associated with receiving a delayed outcome (e.g., by having to wait for the delayed outcome), but delay is not the only cost associated with receiving an outcome. For example, probability and effort have been examined with tasks that have used a similar structure to those assessing delay discounting. Thus, *probability discounting* refers to the process by which an outcome loses value as the odds against its receipt increases (Rachlin et al., 1991), and steep probability discounting reflects a relative preference for smaller, certain rewards (i.e., less risk-taking, more risk aversion). *Effort discounting* refers to the process by which an outcome loses value as the effort required to earn it increases (Białaszek et al., 2019; Mitchell, 2004), and steep effort discounting reflects a relative preference for smaller, easier rewards (i.e., more effort aversion). There is considerably less known about effort discounting than about delay and probability discounting. This may be partly attributable to, as Pinkston and Libman (2017) noted, research manipulating effort requirements must consider that effort has several dimensions, including intensity and duration, and potential differences in its *aversive* effects (i.e., effort is not always aversive per se).

Research comparing delay and probability discounting in the same participants has suggested both can be described using the same equation, suggesting to many that a single-process is operating for both (e.g., Green & Myerson, 2013; Johnson et al., 2020; Rachlin et al., 1986; but also see Killeen, 2022). One way to compare discounting tasks has been to examine correlations between the degree of discounting on these tasks. However, this strategy has led to widely differing correlations between delay and probability discounting being reported. Two common study differences that may contribute to the lack of consensus include different study populations and different measures of the degree of discounting. Comparing studies by Mitchell (1999) and Białaszek et al. (2019) provides a clear example of these mismatches. Mitchell (1999) reported statistically significant, moderately positive correlations between delay and probability discounting gradients in regular smokers and never smokers. In contrast, Białaszek et al. (2019) did not find significant correlations between the two discounting tasks using the area under the discounting curve (AUC) values in healthy participants. However, both Mitchell (1999) and Białaszek et al. (2019) reported significant, moderately positive correlations between effort and probability discounting. Białaszek et al. (2019) also reported moderately positive significant correlations between delay and effort discounting, whereas Mitchell (1999) did not.

As noted above, one reason for the discordant results may be a difference in the measures used to examine the degree of discounting: gradients versus AUC. When using the gradient, studies have often just fitted a single function to the data from all types of discounting, without asking whether the same mathematical model fits the data equally well. In this study, we assessed three commonly used functions to determine which provides the best description of choice for a delay, probability, and effort discounting tasks. First, the hyperbolic model has been used extensively to describe delay and probability discounting (Mazur, 1987; Rachlin et al., 1991) as well as in a few studies with effort discounting (Mitchell, 1999, 2004).

1 $$V=\frac{A}{1+ bX}$$

where *V* represents the subjective value of an outcome, *A* represents the amount of the outcome, *X* can be the delay or odds against or effort to receiving the outcome (*cost*), and *b* represents a free parameter indexing the degree of discounting. Second, the Rachlin (2006) hyperboloid model has shown an adequate fit to the choice data obtained from humans on discounting tasks (e.g., Franck et al., 2015; Young, 2017):

2 $$V=\frac{A}{1+b{X}^s}$$

where the parameters are the same as in Equation 1, and *s* represents a second free parameter indexing the scaling of delay/probability/effort. Both the hyperbolic and hyperboloid functions assume a convex shape of the discounting curve, which tends to underestimate reward value for lower delay/probability/effort levels.

Third, in effort discounting, power functions have also been used to describe discounting (Białaszek et al., 2017):

3 $$V=A-b{X}^s$$

where the parameters are the same as in Equation 2. However, this function produces a concave fit distinguishing it from the two previous models. The concave function tends to overestimate reward value for higher delay/probability/effort cost levels. To our knowledge, only the study by Białaszek et al. (2017) reported that the power function was the best-fitting model for effort discounting, compared to the hyperboloid models and the hyperbolic function. However, the majority of effort discounting studies have not examined the power function. We selected this model as the third candidate to examine in our study because additional research is needed to assess whether the power function provides the best description of data generated from effort discounting tasks, and the adequacy of its fits for delay and probability discounting.

One important feature of delay discounting is its stability over time (test–retest reliability), assessed by having a participant complete the discounting task in the same context on at least two occasions (see Odum et al., 2020). A stable state is “one in which the behavior in question does not change its characteristics over a period of time” (Sidman, 1960, p. 234). This definition does not differentiate between two types of stability. First, *relative* stability, which refers to whether discounting changes in a similar way across people (i.e., correlations across time). Second, *absolute* stability, which refers to whether rates of discounting alter across time points (i.e., means across time). With respect to relative stability, delay discounting shows moderate-strong stability in humans with two (*r* ≥ .70; Matusiewicz et al., 2013; Ohmura et al., 2006; Smits et al., 2013) and three time points (e.g., *r* ≥ .57; Kirby, 2009; Xu et al., 2013). However, data from Matusiewicz et al. and Smits et al. suggested that the type of outcomes (i.e., hypothetical, potentially real or experiential) might affect the relative stability of discounting measures. Other studies have examined the stability of probability discounting and also reported moderate-strong levels of stability *r* ≥ .54–.76 after a 1-week period (Matusiewicz et al., 2013), 3-month period (Ohmura et al., 2006), and 4-month period (Peters & Büchel, 2009). Fewer studies have reported on absolute stability, and to our knowledge, no study has examined the absolute or relative stability of effort discounting.

Studies that have examined the *relative* stability of the discounting measures have most commonly examined correlations between AUC across time points (Anokhin et al., 2015; Ohmura et al., 2006). Martínez-Loredo et al. (2017) used the intraclass correlations coefficient (ICC) to assess the absolute agreement or internal consistency of the observations across time. The ICC had served as a measure of reliability. The *absolute* stability is usually explored using paired *t*-test (if 2 time points) or with repeated measures ANOVA (if > 2 time points). Unfortunately, there are drawbacks to using these frequentist analysis procedures, including their treatment of missing data and focus on rejecting a null hypothesis of no differences. A Bayesian approach, in contrast, provides us with an index of the strength of evidence for the null and alternative hypotheses based on prior evidence and the current observed data (Young, 2019). Thus, to assess whether the degree of discounting is similar between time points for the three discounting tasks we adopted a Bayesian approach.

In summary, our study had two main aims. Aim 1 was to assess the steady-state/stable characteristics of choice patterns in delay, probability, and effort discounting tasks in humans. To do this, we first assessed discounting using an adjusting amount procedure (Du et al., 2002) on each of three time points. Because of uncertainty about the best index of discounting (see Aim 2), we then calculated the AUC for each task on each time point. Based on prior research (e.g., Kirby, 2009; Ohmura et al., 2006), we expected that delay and probability discounting would be stable, in relative and absolute terms, across all time points. In the absence of prior evidence, we had no predictions for effort discounting. Further, we explored the extent to which variability in delay, probability, and effort discounting could be attributed to between- and within-subject differences using a similar approach to prior studies in using rats (Haynes et al., 2021). Aim 2 was to determine whether individuals behaved similarly on the difference discounting tasks using an AUC analysis and, in particular, whether the same mathematical functions could describe delay, probability, and effort discounting equally well.

## Method

### Participants

Twenty-three undergraduate Mexican students (8 male, 15 female) were recruited from a university in Mexico City as a convenience sample. All were between 18 and 22 years old (*M* = 20.48; *SD* = 1.08) and an average of 1.64 m tall (*SD* = 0.06). Only volunteers with a zero or *low* probability of substance use problems were accepted, as assessed using the World Health Organization-ASSIST v3.0 (Henry-Edwards et al., 2003; Linaje & Lucio, 2013). This was expected to reduce sample heterogeneity because there is compelling evidence that participants with substance use or abuse show steeper delay discounting than the controls (Amlung et al., 2017; Bickel et al., 2012). We also required that participants did not report any psychiatric diagnosis nor use any psychiatric medication. Those were the only inclusion and exclusion criteria. Participants were paid MXN$100 (US$4.99) for their participation using Amazon gift cards or recharge cell phone minutes, provided at the end of the second experimental session. They also received course extra credit for participation. All participants provided informed consent prior to participating.

### Apparatus

Sessions were conducted online and participants used their own computers with either Windows10® or macOS® operating systems. AnyDesk®, an open-access application, was used to establish the remote connection between each participant and the laboratory research computer (macOS® Catalina version 10.15.6) so that participants could respond with their own keyboard. All tasks were programmed in Python through an open-access application, OpenSesame® version 3.3.5 (Mathôt et al., 2012).

### Recruitment Survey

We used the Alcohol, Smoking and Substance Involvement Screening Test (World Health Organization-ASSIST v3.0; Henry-Edwards et al., 2003). Individuals report lifetime and 3-month use of a variety of substances and ASSIST evaluates whether they have a low, moderate, or high risk of substance use problems based on their pattern of use. We used the adapted version of ASSIST by Linaje and Lucio (2013) for Mexican young people.

### Procedure

A longitudinal within-subjects design was used. Due to COVID-19 pandemic restrictions, all interactions between the research team and participants occurred remotely using Zoom®, and all sessions were conducted individually. Recommendations provided by the American Psychological Association were used to maintain confidentiality while conducting online sessions (American Psychological Association, 2020).

Participants completed four online sessions on separate days: a screening and informed consent interview, followed by three experimental sessions. During the screening and informed consent interview, participants completed a screening questionnaire that recorded demographics characteristics and substance use history (World Health Organization-ASSIST v3.0). If participants met inclusion criteria (age and zero/low substance use risk), they were provided more details about the study requirements and completed the informed consent process. This interview required approximately 25 min. In the three experimental sessions, the participants performed three computer tasks assessing discounting (described below).

At the beginning of the first experimental session (time point 1), participants completed a calibration task with the help of the researcher overseeing the session, so that physical effort cost levels in the effort discounting task could be individualized. Our calibration task aimed to individualize the effort requirements in an analogous way to procedures adopted in other studies (e.g., Mitchell, 1999, 2004; Sofis et al., 2017), and allowed us to avoid the assumption that all people respond to specific effort costs in the same way. The physical effort required was a specific number of steps. To identify this number, participants were asked to identify a flat, obstruction-free, 3–6 m surface on which they could walk. The researcher showed participants a prerecorded video in which a person demonstrated walking rhythm and body position to walk during the calibration task. After that, participants watched the video again and were asked to count the number of steps to verify that they understood the instructions. Then, participants put a webcam in a position to enable the researcher to observe the participant’s performance and ensure they conducted the steps correctly in each of the 6-min of the task. The researcher used a chronometer and participants were notified about when to start and finish each minute. This stage was used to shape the walking and to offer the same instructions to participants. Later, participants were instructed to walk similarly and count the number of steps as follows (all instructions were provided in Spanish but English translations are provided):

You will walk for a total of 6 min. You have to walk as fast as possible, no running, no jumping. For each minute, you must count the number of steps you have taken. I will tell you when to begin and end with the instructions "Go ahead" and "Stop." At the end of each minute, when you have stopped, you must report the number of steps taken. There will be a new count for each minute. The plan is to calculate the mean number of steps you are able to do in a minute.

The calibration test was not performed for the second and the third experimental sessions. Rather, the same mean number of steps was used for all three time points to remove this as a source of within-subject choice variability between sessions.

After completing the calibration task on the first experimental session, and at the beginning of the second and third sessions, participants made a second remote connection to the researcher’s computer using AnyDesk®. This software allowed participants to view and complete discounting tasks, but the researcher controlled all the procedures and participant responses were downloaded to the laboratory computer; participants did not have access to the software configurations. During the discounting tasks, the researcher’s webcam was off to reduce distraction, but the participant’s webcam and microphone were on so that the researcher could verify responses were occurring from the participant.

On all three experimental sessions, the participants read the following instructions to familiarize/refamiliarize them with the discounting tasks:

You will respond to a series of options to earn rewards. There are no correct or incorrect choices. There is no time limit to respond. You will not actually receive the rewards during the task nor at the end of the session, but we ask you to respond as if you were going to win them. The gains are not cumulative across the alternatives. Also, each choice you make is independent of the other choices. Choose the option that you prefer and not the one that someone else would choose. Respond according to your preferences today. Avoid responding based on the past or future. The options will be displayed on the screen. To make your choices: use the Z keyboard to select the options on the left side. Use the M keyboard to select the option on the right side.

Then, participants completed six forced-choice trials, where alternatives were presented in the same way as in the subsequent discounting tasks but participants were told which choices to make. Afterwards, participants were prompted to ask the researcher if they had any questions. Then, the first discounting task began. There was no break between tasks.

For the three discounting tasks, an adjusting amount procedure was used to obtain indifference points (IPs) across the different cost levels (Du et al., 2002). Each discounting task included 30 choice trials: six choices at each of five delays, six choices at each of five probability levels and six choices at each of five effort cost levels. Thus, each discounting task yielded five IPs (one for each cost level). The order of task presentation (i.e., delay, probability, and effort) was random, but all 30 choice trials within each discounting task were presented before the next task began. Within each discounting task, the order in which the five delays/probabilities/effort levels were presented was random, but all six choice trials for each level were presented before the next level began. On each choice trial, the participants considered two alternatives: (1) a smaller amount of money available immediately/for sure/with a low effort exertion requirement; or (2) a larger amount of money available after a delay/with some probability/with a high effort exertion. The location of the two alternatives was randomly assigned to the right and left of the computer screen from trial to trial. On the first-choice trial of each level, the larger amount of money was always MXN$3,000 (US$147 at that time), and the smaller amount was half that (MXN$1,500).

The following description uses the delay discounting task as an example to illustrate the procedure for all tasks. On the first-choice trial in the delay discounting task, a participant might be asked to choose between $3,000 after a delay (e.g., 2 months) or $1,500 received immediately. For the subsequent five trials, the amount of the immediate reward was adjusted based on participant choices following the algorithms provided by Du et al. (2002), whereas the delay to $3,000 was maintained at 2 months. This procedure was repeated until six choices were made for each delay level (i.e., each delay varied within a block of six trials). The amount of the immediate reward on the sixth and final choice at a specific delay level was coded as the IP. IPs represent the amount of the smaller, sooner reward that is considered subjectively equal to the amount of the delayed alternative. Immediate amounts were rounded to the nearest whole number to avoid a harder processing of the amounts (e.g., Kallai & Tzelgov, 2014).

#### Specific Task Instructions

For the delay discounting task, participants read the following instructions “In this task you will have to choose between immediate or delayed rewards, for example, would you rather earn $10 NOW or $20 AFTER a delay (1 day)?” We used different delays and amounts of money for the instructions to avoid a bias before the trials. The researcher asked to participants if they had any questions and pointed out the relevant portions of the instructions when answering questions. The five delay levels used were 2 weeks, 2 months, 6 months, 1 year, and 3 years.

Before beginning the probability discounting task, participants read instructions clarifying the concepts about certainty and chance of receiving a reward (Secretaría de Educación Pública, 2011), and had an opportunity to ask questions about these concepts. Then, the participants read the following instructions, “In this task you will have to choose between certain or risky rewards, for example, would you rather earn $10 for sure or $20 with 20% of chance?” The five probability levels were 90, 75, 50, 25, and 10% (0.11, 0.33, 1, 3, and 9 odds against receiving the reward).

For the effort discounting task, participants read the following instructions “Imagine walking at the same speed as you did the step test that you performed in the first session. In that test, your average of number of steps was [*insert average number of steps*] in 1 min. In this task you will choose between rewards for walking fewer steps or walking more steps, for example, would you rather earn $10 after walking 78 steps or $20 after walking 120 steps?” The *fewer steps* alternative always used the mean number of steps in 1 min taken by the specific participant, whereas the five *more steps* effort levels corresponded to the number of steps the participant would take in 10, 20, 60, 90, and 120 min at that walking speed. Thus, although the number of steps was individualized, the underlying durations of walking was the same for each participant and was used as the cost level for analyses of IPs and AUC calculations. The specific increments of minutes selected for the study was based on several health advertisements in Mexico used to encourage walking and reduce sedentarism. No walking duration was specified in the instructions to the participants. A nonzero number of steps was used for the fewer steps alternative to distinguish it from the immediate reward alternative in the delay discounting task (e.g., Mitchell, 1999).

#### Session Timing

Data collection was conducted between August and December 2020, when colleges closed due to the COVID-19 pandemic and students worked from home. Sessions took place from between 8 am–4 pm (UTC-6) for each participant. Participants were asked to return for the second experimental session 2 weeks after the first session, and for the third session, 2 weeks after the second session (e.g., Xu et al., 2013). The participants were contacted by text message to confirm and remind them of session appointments. Most of the participants met the interval appointment. Four participants completed the second and the third time points a couple of days later than the expected interval (2 or 4 days).

### Data Analysis

The model fits, ICC, and all graphical analyses were conducted using *R*® (R Core Team, 2022) and *RStudio*® as the development environment (RStudio Team, 2020). The packages/functions used in the analysis are noted where relevant (Online Resource 1). Microsoft Excel® (version 16.16.27) was used to facilitate the calculation for AUC-values (Online Resource 2). We used JASP® (version 0.16.3), an open-source software, to perform the Bayesian analyses (e.g., Vincent, 2015).

#### Relative and Absolute Stability

Aim 1 of the present study was to assess the stability of choice patterns across the three time points for delay, probability, and effort discounting. To do this, we selected an aggregate measure of discounting that is neutral with respect to mathematical function: AUC (Myerson et al., 2001). The AUC is derived by summing the area of each trapezoid formed by two adjacent IPs and their corresponding levels of delay, probability (odds against), or effort: *x*_2_ – *x*_1_ [(*y*_1_ + *y*_2_) / 2]. The values *x*_2_ and *x*_1_ are the normalized levels of cost, and *y*_1_ and *y*_2_ are the normalized IPs at those levels. We also calculated the ordinal AUC (AUC_*ord*_), because this improved normality and homoskedasticity (Borges et al., 2016), by replacing the numerical values of each cost level in the discounting task with integers from 1 through 5 (e.g., the first delay, 2 weeks, is recoded as “1,” the second delay, 2 months, as “2,”). The AUC_*ord*_ ranges from 0 to 1, with lower AUC_*ord*_ values indicating greater *impatience*, more risk aversion, or more effort aversion. Because the results using both traditional and ordinal AUC were nominally different, only analysis involving AUC_*ord*_ are reported.

The *relative* stability was assessed in two ways. First, we calculated Bayesian Pearson product–moment correlations using the AUC_*ord*_ values to examine the test–retest reliability between each time point for each task (Online Resource 3). Values provided in Taylor (1990) were used to interpret the strength of the correlation coefficient: *r* ≤ .35 are weak, ranges between .36 and .67 are moderate, ranges between .68 and .9 are strong, and ≥ .9 are very strong correlations. Values provided in Doorn et al. (2021) were used to interpret the Bayes Factor (BF_10_) values; an index to quantify the weight of evidence for the competing null (*H*_0_) and alternative (*H*_1_) hypotheses. In these correlational analyses, the *H*_0_ is that there is no relationship between the AUC_*ord*_ for a given pair of time points, whereas the *H*_1_ is that there is a relationship. BF_10_ value ranges between 0.3 and 3.0 indicate weak evidence for either *H*_0_ or *H*_1_, ranges between 3 and 10 indicate moderate evidence for the *H*_1_, and BF_10_ > 10 indicates strong evidence for the *H*_1_.

Second, we standardized the AUC_*ord*_ values by converting them to *z*-scores and used a two-way mixed-effects model with absolute agreement to calculate the ICC for theses standardized AUC_*ord*_ values across time for each task (e.g., Martínez-Loredo et al., 2017). The ICC is suitable for testing test–retest reliability when there are more than two repeated measures over time (Koo & Li, 2016), allowing us to conducted a separate ICC for each task using all three time points for each participant. The ICCs were calculated with the *irr* package (Gamer et al., 2010). Values less than 0.50 indicate poor reliability over the three time points, values between 0.50 and 0.75 indicate moderate reliability, values between 0.75 and 0.90 indicate good reliability, and values greater than 0.90 indicate excellent reliability. The ICC has also been used to study the variability attributed to state- and trait-like differences in rats (e.g., Haynes et al., 2021), and we used these same ICC values to explore the extent of variability in delay, probability, and effort discounting that could be attributed to between- and within-subject differences. In this context, ICC > .5 indicate that the AUC_*ord*_ differs more between-subjects than within-subjects, i.e., is relatively more consistent across time points. This is interpreted as reflecting trait-like differences. ICC < .5 indicate that AUC_*ord*_ differs more within-subjects, i.e., is relatively less consistent across time points within-subjects. This is interpreted as reflecting state-like differences. Although ICCs are considered measures of trait and state variability (Merz & Roesch, 2011), they should be interpreted cautiously because ICCs do not allow us to identify the specific sources of between-subject variability or within-subject variability.

As supporting analyses, we conducted three separate Bayesian repeated measures ANOVAs with the IPs to explore the *absolute* stability across time points for each discounting task. That is, for each task, we performed a separate repeated measures ANOVA with the cost levels as the within-subject factor and the three time points as the between-subject factor. All ANOVAs incorporated random intercepts and slopes (Online Resource 4) and examined the weight of evidence for whether there was no effect of cost level or time point on the IPs (*H*_0_) or one or both of these variables affected the IPs (*H*_*1*_).

#### Evaluation of a Common Process for Discounting

Aim 2 was to determine the extent to which choice behavior on the three discounting tasks was similar and could be described by the same mathematical equation, potentially implying a common process. To address this aim we conducted two analyses. First, to Bayesian correlations between the AUC_*ord*_ values for each task at each time point were used to explore whether participants responded in a similar way among the discounting tasks, i.e., someone who discounted delayed rewards to a large degree also discounted effort-requiring rewards to a large degree, etc. (Online Resource 3). We used the same criteria to evaluate the evidence for the *H*_0_ and *H*_1_ hypotheses as used in the relative stability analyses described earlier. Strong positive or strong negative correlations were viewed as consistent with a common process (Johnson et al., 2020). On the other hand, weak or moderate positive or negative correlations between the AUC_*ord*_ values for each pair of tasks were viewed as consistent with the operation of different choice processes.

Second, we examined whether the same mathematical function could describe delay, probability, and effort discounting over time. For each task and each time point, we used the *nlmrt* nonlinear regression package (Nash, 2016) to fit the three discounting models (Eqs. 1, 2, and 3) to the median IPs. We used the Second-order Akaike Information Criteria (AICc) for model comparisons because accounts for the best-fitting model for small sample sizes. We used the AICc differences (Δ_*i*_ AICc) for fit comparisons and ranking of candidate models (Burnham & Anderson, 2004):

4 $${\Delta }_i={\textrm{AICc}}_i-{\textrm{AICc}}_{\textrm{min}}$$

where AIC_*i*_ is the AICc for the *i*^th^ model and AICc_min_ in the minimum of the AICc among all the models. Models differing from the AICc_min_ model by ≤ 2 have substantial support, those for which 4 ≤ Δ_*i*_ ≤ 7 have less support, and models having Δ_*i*_ > 10 have essentially no support. Thus, the best model has Δ_𝑖_ ≡ Δ_𝑚𝑖𝑛_ ≡ 0. These guidelines have similar counterparts in the Bayesian literature (Raftery, 1995).

## Results

### Relative and Absolute Stability

**Fig. 1:**
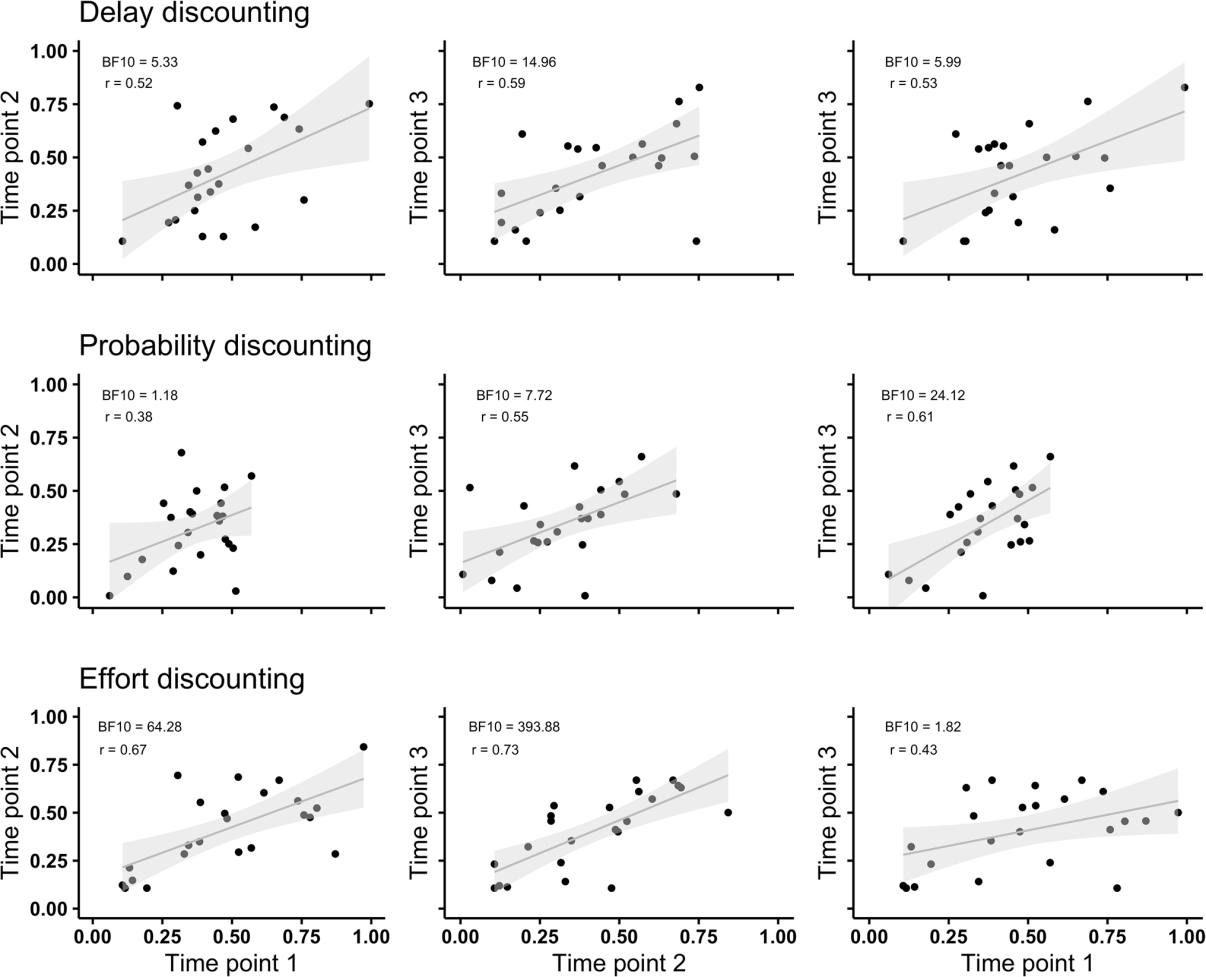
Test–Retest Correlations with 95% Confidence Intervals. *Note.* Circles represent the AUC_*ord*_ values for individuals. The left column shows time points 1 and 2, the middle column shows time points 2 and 3, and the right column shows time points 1 and 3. The top row depicts the test–retest data for delay discounting, the middle depicts the probability discounting time points, and the bottom row depicts the effort discounting data. Each test–retest graph includes its BF_10_ value and the Pearson correlation coefficient

**Fig. 2:**
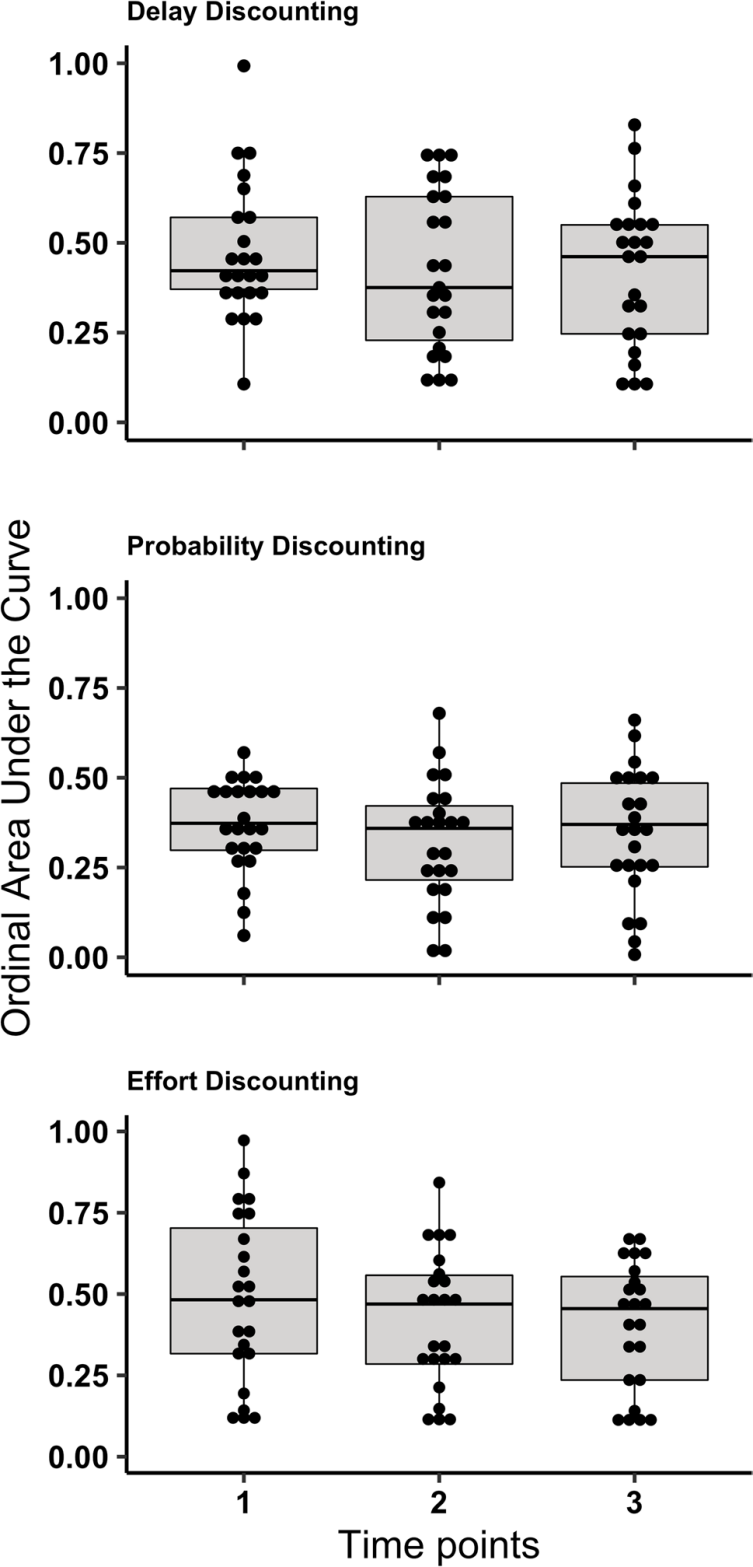
AUC_*ord*_ for Delay, Probability, and Effort Discounting across Time Points. *Note.* Box plots of AUC_*ord*_ values for the participants who completed the discounting tasks across the three time points. The bottom and top of each box represent the 25^th^ and 75^th^ percentiles, the horizontal line within each box represents the group median. The vertical lines extending from the boxes represent the minimum and maximum values that are not outliers. The circle out of the whiskers is an outlier (delay discounting at Time 1). The symbols inside each box represent the individual AUC_*ord.*_

The *relative* stability was assessed first by examining the Bayesian test–retest correlations of AUC_*ord*_ values on pairs of the time points for each task. Figure 1 displays the scatterplots of pairs of time points for each of the three tasks. All correlations were positive. The magnitude of correlations between AUC_*ord*_ values for delay discounting were moderate (range in *r* = .52–.59), as well as for probability discounting (range in *r* = .38–.61), whereas moderate and strong correlations were observed for effort discounting (range in *r* = .43–.73). Bayesian statistics indicated that there was moderate-to-strong evidence for the alternative hypothesis (*H*_1_) that there was a relationship between pairs of time points in delay discounting (BF_10_ ≥ 5.33–14.96). Evidence for a relationship was ranged from weak-to-strong for probability discounting (BF_10_ ≥ 1.18–24.12) and effort discounting (BF_10_ ≥ 1.82–393.88). Time point pairs for which the evidence was weakest or strongest varied. That is, it was *not* the case that correlations were higher and relationships were strongest for consecutive time points, with the time point 1 versus time point 3 showing the least stability. This observation was supported by the ICC analyses, which considered all three time points for each discounting task. These analyses indicated moderate stability of choice patterns for each task. The ICCs for delay, probability, and effort discounting were 0.56 (95% CI [0.31, 0.76]), 0.53 (95% CI [0.27, 0.73]), and 0.62 (95% CI [0.38, 0.79]), respectively. By multiplying the ICCs by 100%, the percentages can be used to examine the extent of variability attributed to trait- and state-like differences in AUC_*ord*_. The 56%, 53%, and 62% values for the delay, probability, and effort discounting tasks indicates that more of the variability in AUC_*ord*_ is attributable to between-subject (trait-like) variability than within-subject (state-like) variability. The ICCs for delay and probability discounting are fairly similar, with a slightly highest ICC for effort discounting. This difference is attributable to the larger variability in the choice patterns between-subjects for the effort discounting task. Figure 2 displays the AUC_*ord*_ values for the three discounting tasks at each time point.

### Evaluation of a Common Discounting Process

We conducted two analyses to determine the extent to which all discounting tasks shared a common process. First, we calculated the correlations between AUC_*ord*_ between pairs of tasks at each time point (see Online Resource 3). Overall, there was strong evidence for the *H*_1_, which stated that there was a relationship between delay and probability discounting, at time point 2 (BF_10_ = 14.13, 95% CI [0.19, 0.78]) and time point 3 (BF_10_ = 37.98, 95% CI [0.27, 0.81]). Both correlations were moderate and positive *r* = .58 and *r* = .63, respectively. These results indicate that individuals who showed high AUC-delay values also tended to show high AUC-probability values. However, support for the alternative hypothesis was weak for all other comparisons (0.3 < BF_10_ < 3).

**Fig. 3:**
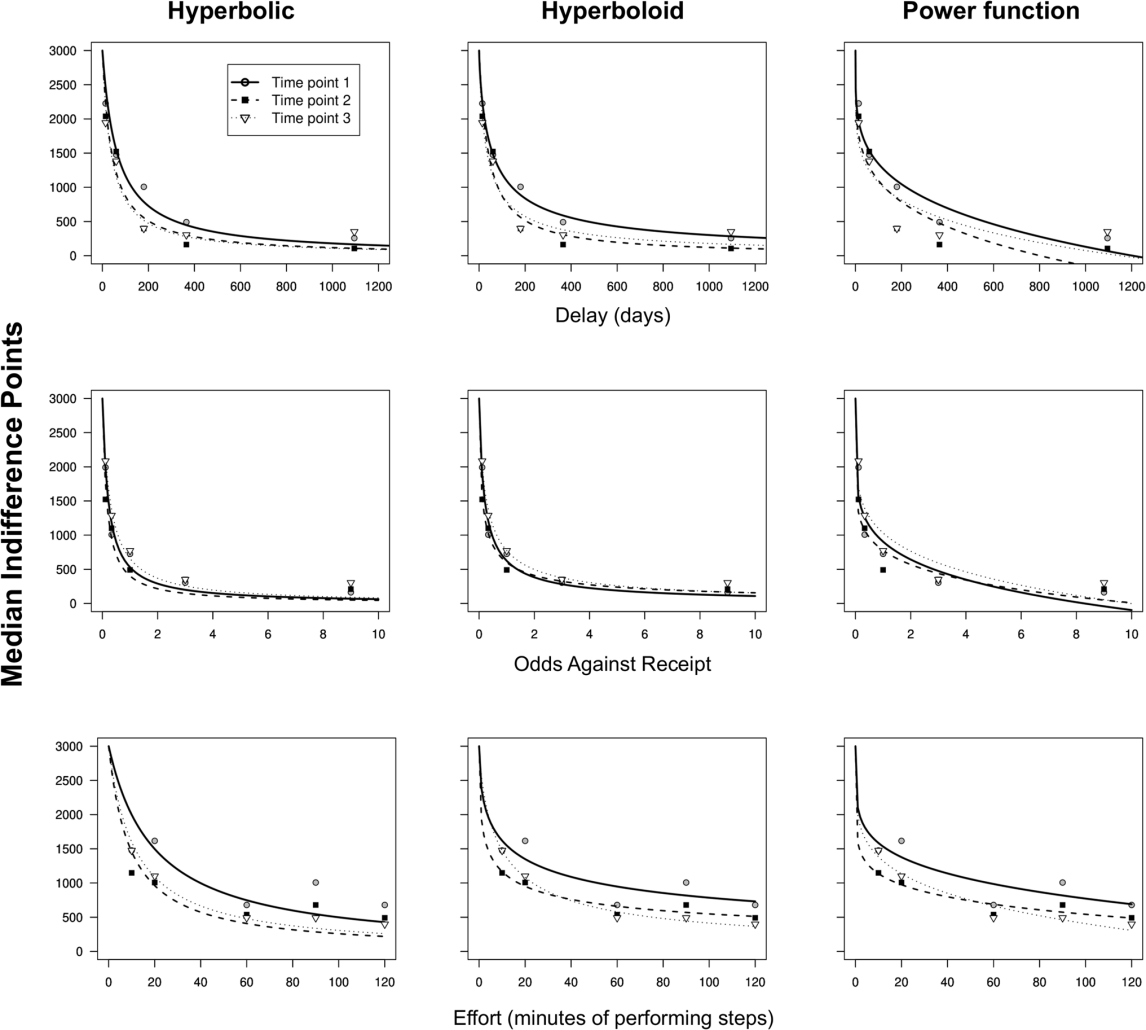
Model-Fitting to the Median Indifference Points across Time Points for Each Task. *Note.* Hyperbolic (first column), hyperboloid (middle column), and power function (last column) model fits to the median IPs among tasks and across time points. The top row shows the fits for the delay discounting task, the middle row shows those for the probability discounting task, and the bottom row, the effort discounting task

We also examined whether the same mathematical function could describe delay, probability, and effort discounting across time. Overall, Table 1 shows that the hyperbolic (Eq. 1) and the hyperboloid (Eq. 2) functions had the lowest AICc values for the median IPs for all three time points, indicating superior fits to the data. There were no instances of the power function providing a comparable fit (Δ_*i*_ AICc > 7). In other words, a convex mathematical form was clearly better able to describe the IPs obtained from the delay and probability tasks. The data were more mixed for the effort discounting IPs. The hyperboloid and power functions provided comparable fits for IPs at time points 1 and 2, whereas the hyperbolic and hyperboloid were indistinguishable for time point 3 IPs. However it is work noting that there was limited support for the hyperbolic at time points 1 and 2 (Δ_*i*_ AICc ≤ 7), and the power function narrowly missed that cut off for time point 3 ((Δ_*i*_ AICc = 7.38). In summary, there was not a unique model which best described IPs for all time points for each task. Figure 3 shows the three equations fitted to the median IPs for each task on all three time points. By visual inspection, the three tasks show a convex form of the discounting patterns.

**Table 1:** Comparison of AICc Values for the Three Mathematical Models with the Median Indifference Points

Task	Time point	AICc			Δ_*i*_ AICc		
		Eq. 1	Eq. 2	Eq. 3	Eq. 1	Eq. 2	Eq. 3
Delay	1	**69.93**	68.79*	78.63	1.14		9.84
	2	71.90*	78.56	83.91		6.66	12.01
	3	72.19*	77.44	83.63		5.25	11.44
Probability	1	68.31*	73.27	82.06		4.96	13.75
	2	70.98	68.00*	77.91	2.98		9.91
	3	67.86*	69.91	82.1		2.05	14.24
Effort	1	77.73	**74.57**	74.47*	3.26	0.10	
	2	75.21	69.79*	**70.47**	5.42		0.68
	3	**66.30**	64.76*	72.14	1.54		7.38

*Note.* Eq. 1 is the hyperbolic. Eq. 2 is the hyperboloid. Eq. 3 is a power function

*The best model for each time point is that with the smallest AICc (also indicated with bold font). But models with Δ_*i*_ AICc < 2.0 cannot be statistically distinguished from the model with the smallest AICc value are also indicated with a bold font

## Discussion

The Aim 1 of the present study was to assess the steady-state characteristics of choice patterns across three time points for delay, probability, and effort discounting tasks with hypothetical rewards in humans. Overall, our results replicated the previous findings of positive and moderate *relative* stability in delay discounting (e.g., Kirby, 2009; Martínez-Loredo et al., 2017), as well as in probability discounting (e.g., Matusiewicz et al., 2013; Ohmura et al., 2006). Our conclusions about stability are supported by the ICC scores, which indicated there was more variability between-subjects compared to within-subject differences across time points. The evidence for the *absolute* stability (see Online Resource 4) indicated a negligible role for time point on IPs for all three discounting tasks, and that the levels of delay, probability, and effort were the primary determinants of the IPs. This result for delay discounting was partially consistent with prior findings (e.g., Kirby, 2009; Xu et al., 2013). The use of the Bayesian approach to determine that there is only weak evidence to support an interaction between the IPs and the three time points in delay discounting extends the results from Xu et al. (2013) in young adults, who explored the absolute stability across three time points with the frequentist approach. This consistency is important because it should reduce the concerns about using different statistical approaches. It should also increase confidence in using Bayesian analyses, which provide information about the degree of support to both the null and the alternative hypothesis, rather than the dichotomous decisions process based on the *p*-values in frequentist analyses. To our knowledge, no previous study has explored the absolute and relative stability of effort discounting in humans. Thus, our study extends results indicating that the delay and probability are fairly stable to effort discounting, at least over the 4-week period of the current study. We suggest that future studies should explore longer periods.

As part of the evaluation process for our examination of stability we used the ICC scores. The data suggested that variability in AUC_*ord*_ values for each of the discounting tasks was associated with trait-level rather than state-level differences. Unfortunately, this analysis does not permit us to identify the sources of between and within-subject variability. There are several potential factors that may contribute to the general incidence of variability across all tasks. Perhaps the most salient is that our study was conducted during the COVID-19 pandemic during the early phases when lockdowns and self-isolation occurred in Mexico City. Romanowich and Chen (2021) found low test–retest reliability in delay discounting immediately after the environmental disruption by COVID-19, suggesting that major environmental disruptions might negatively affect the stability of discounting measures. This may have occurred because the COVID-19 pandemic may have altered the value of rewards when individuals were socially isolated and less certain about the future (Romanowich & Chen, 2021). Thus, our stability estimates may be lower than would have been obtained during non-COVID times.

Our Aim 2 was to evaluate evidence that a common process underlies the three discounting tasks. The evidence examined included the correlations between AUC_*ord*_ values for each task at each of the three time points, and similarities in the mathematical functions that best described the IPs. On balance, the positive correlations were larger between AUC_*ord*_ values for delay and probability discounting than for any comparisons with effort discounting, with strong support for this conclusion from the Bayesian analyses. Thus, there appears to be some commonality in the processes underlying choices in the delay and probability tasks but not with the effort discounting task, despite the small positive correlations between its AUC_*ord*_ values with those of the other tasks (Online Resource 3). These general conclusions were supported by our finding that the hyperbolic and hyperboloid functions showed the best-fitting for more instances to the delay and probability discounting tasks across time points, whereas the best fitting equations for IPs from the effort discounting task included the power function. The result for the delay and probability tasks is partially consistent with prior evidence (e.g., Ohmura et al., 2006). However, Ohmura et al. used *R*^*2*^ values to compare the mathematical functions and drew conclusions based on which had the higher scores. Use of *R*^*2*^ rather than AIC, AICc (used in our study) or the Bayesian Information Criterion (BIC) is an active area of discussion among researchers. For example, Johnson and Bickel (2008) warned about using *R*^*2*^ values to compare delay discounting models as they suggest there is overfitting for models with two or more free parameters and correlations between *R*^*2*^ and discounting parameters. It is to be hoped that future discussions will achieve a consensus about methods to identify the best-fitting model.

We found that the hyperboloid function fit the effort discounting task IPs well for two time points. This is inconsistent with prior evidence indicating the superiority of the power function (e.g., Białaszek et al., 2017) and our expectation that the shape of the effort discounting function would be more concave (i.e., power function) rather than convex. However, the visual inspection of IPs data and Δ_*i*_ AICc values indicated that the effort discounting choice patterns were convex, similarly to those for delay and probability discounting. However, the shape of the effort discounting function seems to vary across studies. For example, Mitchell (1999) found a shallow effort discounting curve and a good fit to the hyperbolic model. Białaszek et al. (2017) also found a similar shallow pattern, but the power function fitted their data better rather than any tested convex models. In both studies, effort was defined as exertion of force using a hand dynamometer (i.e., Maximum Voluntary Contraction). In contrast, when effort is defined as number of activities or the number of responses during a specific period, the shape of the curve seems more convex, as in our study, and in a study reported in Ostaszewski et al. (2013). We suggest that future research should explore whether the mathematical form of effort discounting data depends on the definition of effort requirements (i.e., force vs. number of responses or duration of responding).

Despite the clear and intriguing differences in choices from the discounting tasks revealed in this study, we also identify some study limitations. First, our study used hypothetical cost levels and reward outcomes for all tasks. There is some data suggesting the use of hypotheticals does not result in different qualitative results (Lawyer et al., 2011; Madden et al., 2003). However, this is not the case for all studies. For example, Hinvest and Anderson (2010) reported that the use of real outcomes was associated with significantly decreased impulsive choices in delay discounting compared to hypothetical outcomes. Also, Matusiewicz et al. (2013) reported inconsistent results about the stability of delay discounting with hypothetical and potentially real outcomes. Possibly consistent with this hypothetical–real concern is that the ICC score was higher for the effort discounting task, reflecting more variability between-subjects. Although speculative, this may be attributable to the pre-exposure to the effort requirements during the calibration task (Eisenberger, 1992), which could have altered participants’ ability to imagine the hypothetical effort requirements. The larger between-subject variability in ICC may reflect the individual differences in performance on the calibration task, which we conducted to ensure the number of steps in specific time periods were equated for each participant. The analysis revealed that there was relatively weak evidence for relationship between the AUC_*ord*_ for the effort discounting task at any time point and the mean number of steps taken during that task (Online Resource 5). Future studies should compare performance on effort discounting tasks when participants are exposed to a calibration task that allows them to experience the effort requirements compared with wholly hypothetical or real requirements and outcomes.

A second limitation related to the use of a nonzero effort requirement for the small reward, compared to the zero delay (immediate smaller reward) and zero odd-against/probability =1 (smaller reward for sure) in the other tasks. Prior research has indicated that nonzero delays in delay discounting tasks do not alter the discounting function (e.g., Green et al., 2005; Mitchell & Wilson, 2012), but it is unclear whether this is the case with probability discounting. Thus, although we do not rule out the possibility that the current manipulation could influence effort discounting differently from the other two tasks, we could not identify any data that would indicate the relative relationships across time points or between tasks would be systematically disrupted. However, future research to evaluate the effects of nonzero values would be useful.

A final limitation associated with the effort discounting task was that we created the different levels of effort by multiplying the steps completed in 1 min, assessed the calibration task, by specific numbers of minutes (10, 20, 60, 90, and 120 min). In the natural environment, walking pace might be expected to vary limiting our ability to translate to the number of steps to match the duration of walking. Individuals’ recognition of this change in walking pace might have contributed to the high levels of between subject variability observed in this task. Again, we suggest future research exploring the effects of real versus hypothetical requirements would be useful, especially in the realm of physical effort discounting.

In conclusion, although we recognize that the study has some limitations, the data indicate that an individual’s effort discounting is stable and reliable over approximately a month, similarly to delay and probability discounting. Further, the choices made in the three discounting tasks are only modestly similar, which supports the conclusion that the choice process in effort discounting is dissimilar to that of delay and probability discounting, and that discounting processes are a function of *cost* type (Białaszek et al., 2019).

## Data Availability

The datasets generated during and/or analyzed during the current study, as well as the code in R® for conducting the analyses, are hosted on Open Science Framework: https://osf.io/3bkjq/?view_only=ee27660653b244e2be28c498a99a7918 and available from the corresponding author.

## Declarations

The current manuscript has not been previously published and has not been and will not be submitted elsewhere during the review process. The current manuscript has been approved by all co-authors.
